# Familial adenomatous polyposis-associated desmoid disease: comprehensive review

**DOI:** 10.1093/bjsopen/zrag089

**Published:** 2026-09-29

**Authors:** Benjamin Zare, Susan Clark, Palma Dileo, David Liska, Andrew Latchford

**Affiliations:** St Mark’s Centre for Familial Intestinal Cancer, St Mark’s Hospital, London, UK; Department of Surgery and Cancer, Imperial College London, London, UK; St Mark’s Centre for Familial Intestinal Cancer, St Mark’s Hospital, London, UK; Department of Surgery and Cancer, Imperial College London, London, UK; Sarcoma Unit, University College London Hospitals NHS Foundation Trust, London, UK; Department of Medical Oncology, Istituto Ortopedico Rizzoli, Bologna, Italy; Department of Colon and Rectal Surgery, Digestive Disease and Surgery Institute, Cleveland Clinic Foundation, Cleveland, Ohio, USA; The Sanford R. Weiss, MD Center for Hereditary Colorectal Neoplasia, Department of Colorectal Surgery, Digestive Disease Institute, Cleveland Clinic, Cleveland, Ohio, USA; St Mark’s Centre for Familial Intestinal Cancer, St Mark’s Hospital, London, UK; Department of Surgery and Cancer, Imperial College London, London, UK

**Keywords:** desmoid tumour, desmoid-type fibromatosis, *APC*, active surveillance, tyrosine kinase inhibitor, nirogacestat

## Abstract

**Background:**

Familial adenomatous polyposis (FAP)-associated desmoid disease is a rare but important manifestation of FAP and remains a major cause of morbidity and mortality. In contrast to sporadic desmoids, which most commonly occur in the abdominal wall or extra-abdominal sites, approximately 70% of FAP-associated desmoids arise intra-abdominally, usually within the small bowel mesentery. These lesions can cause bowel and ureteric obstruction, sepsis, intestinal failure, and death. This review summarizes current evidence regarding the pathogenesis, risk factors, diagnosis, surveillance, and management of FAP-associated desmoid disease, and identifies key evidence gaps.

**Methods:**

A literature search was performed in accordance with PRISMA methodology using Medline, Embase, PROSPERO, Cochrane, Campbell, and Joanna Briggs Institute databases. Original research studies, reviews, and guidelines relating to desmoid disease, FAP-associated desmoid disease, and FAP published up to August 2025 were eligible. Data were extracted and synthesized narratively by topic.

**Results:**

Aberrant Wnt/β-catenin signalling resulting from a germline *APC* pathogenic variant and a somatic second hit underpin the development of FAP-associated desmoid disease. Established risk factors include a distal *APC* pathogenic variant, positive family history, and previous abdominal surgery. Diagnosis in patients with established FAP is usually clinicoradiological, and routine biopsy is generally unnecessary, except in select circumstances. Active surveillance is now recommended as first-line management for most patients because many lesions remain stable or regress spontaneously. Surgery is primarily reserved for complications, particularly in small bowel mesenteric disease, owing to substantial operative morbidity and a lack of demonstrated survival benefit from complete resection. Systemic therapy is indicated for progressive or symptomatic disease. Tyrosine kinase inhibitors and the γ-secretase inhibitor nirogacestat have demonstrated efficacy in randomized trials, although patients with FAP-associated disease comprise only a small proportion of enrolled patients. Evidence supporting the use of non-steroidal anti-inflammatory drugs and anti-oestrogens remains limited.

**Conclusion:**

Important evidence gaps remain regarding optimal imaging strategies, predictors of aggressive disease, clinically meaningful trial endpoints, and FAP-specific treatment algorithms. Dedicated studies in genetically defined FAP cohorts are required to improve risk stratification and management.

## Teaser

Familial adenomatous polyposis (FAP)-associated desmoid disease is a major source of morbidity and mortality in FAP, typically arising intra-abdominally and driven by an *APC* constitutional pathogenic variant with dysregulated Wnt/β-catenin signalling. Owing to high operative morbidity and the absence of survival benefit with complete resection, management has shifted away from primary surgery towards active surveillance, reserving operative intervention mainly for complications such as bowel obstruction, ischaemia, or sepsis, particularly in small bowel mesenteric disease; systemic therapies are used for patients with progressive or symptomatic disease. This review summarizes current evidence-based management, as well as highlighting the significant evidence gaps that remain: prognostic markers, imaging strategies, trial endpoints, and FAP-specific treatment algorithms.

## Introduction

Desmoid disease is one of the most important extra-intestinal manifestations of familial adenomatous polyposis (FAP) and remains a significant cause of morbidity and mortality despite advances in surveillance, surgical techniques, and medical therapies. FAP-associated desmoid disease differs from sporadic desmoid disease in its epidemiology, anatomical distribution, biological behaviour, and clinical management. However, the evidence base remains limited, with much of the available literature comprising retrospective studies, small case series, and extrapolation from studies of sporadic desmoid disease. Consequently, variation exists in clinical practice and among published guidelines.

The aim of this review is to summarize current evidence regarding the pathogenesis, risk factors, diagnosis, surveillance, and management of FAP-associated desmoid disease, identify areas of controversy and uncertainty, and highlight priorities for future research.

## Methods

A literature search was performed in accordance with PRISMA recommendations^1^. Medline, Embase, PROSPERO, Cochrane, Campbell, and Joanna Briggs Institute (JBI) Evidence Synthesis databases were searched for original research studies, reviews, and guidelines relating to desmoid disease (including recognized pseudonyms), FAP-associated desmoid disease, and FAP published up to August 2025 (*Supplementary material*). Non-English language publications were excluded. Relevant studies were identified and data extracted using the JBI data extraction tool^2,3^. Findings were synthesized narratively and organized according to key clinical themes.

### Desmoid disease

Desmoid disease (also known as desmoids, desmoid tumours, aggressive fibromatosis, deep fibromatosis, and desmoid-type fibromatosis) is very rare, accounting for 0.03% of all new neoplasms^4^. There is considerable uncertainty as to whether desmoid disease should be classed as a ‘neoplasm’; although desmoids comprise clonal expansions of cell populations (with somatic impairment of Wnt signalling, due to loss of *APC* function in FAP and *CTNNB1* in patients with sporadic disease), they do not metastasise and stop growing in most patients. It may be that desmoid disease should be categorized with the superficial fibromatoses (in which somatic genetic alterations in Wnt signalling have also been identified), to which they are arguably more similar^5–7^. Most desmoids (∼90%) arise sporadically, but patients with FAP are 850- to 1000-fold more likely to develop them than the general population^8,9^, and the lifetime incidence of desmoid disease in patients with FAP is 10–25%^10^.

Desmoids can form masses or sheets/plaques, with the latter often difficult to measure, posing a challenge in radiological assessment for surveillance or treatment response^11^. The cut surface is often whorled and resembles scar tissue^12^. In terms of biology and corresponding behaviour, desmoids remain enigmatic^5^ (*Fig. 1*). Specific histological features include dense, collagen-rich stroma; low-to-moderate cellularity with long fascicles of uniform cells (myofibroblasts: specialized, well differentiated fibroblasts whose phenotype differs morphologically from that of fibroblasts by the expression of α-smooth muscle actin within their cytoplasmic stress fibres); and a lack of malignant features^13,14^ (*Fig. 2*).

**Fig. 1:**
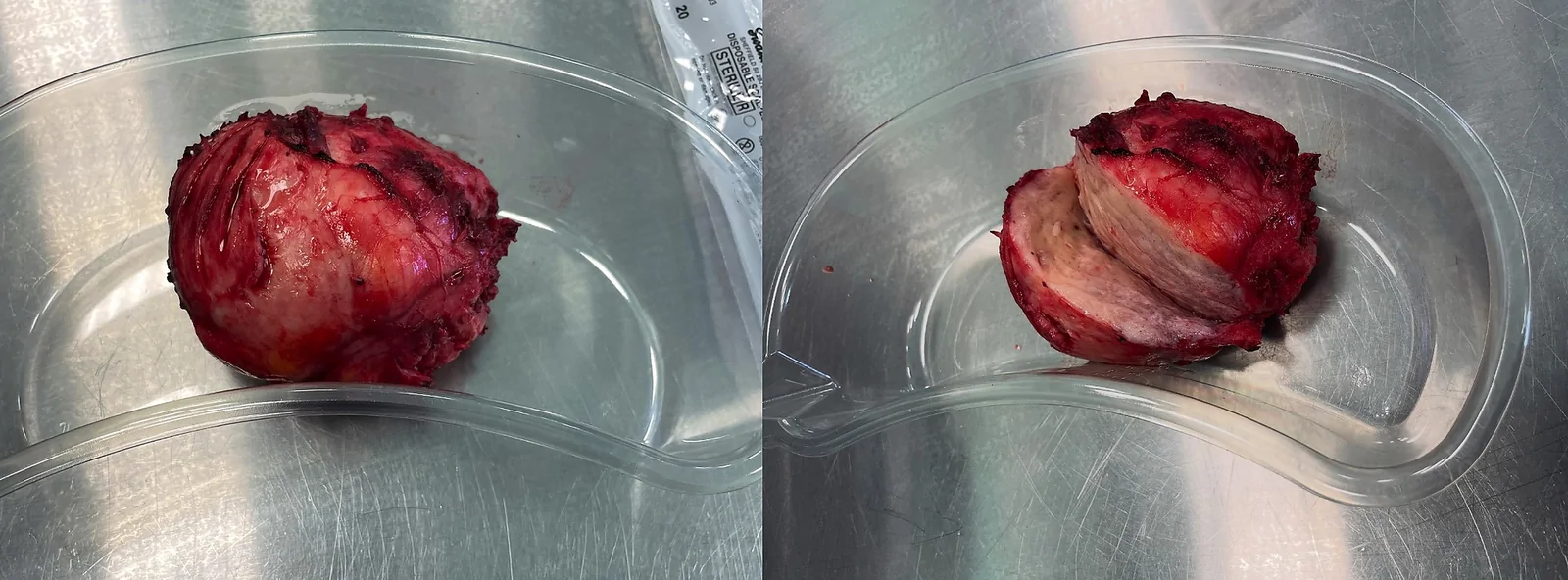
Resected abdominal wall desmoid (left) with cut surface (right)

**Fig. 2:**
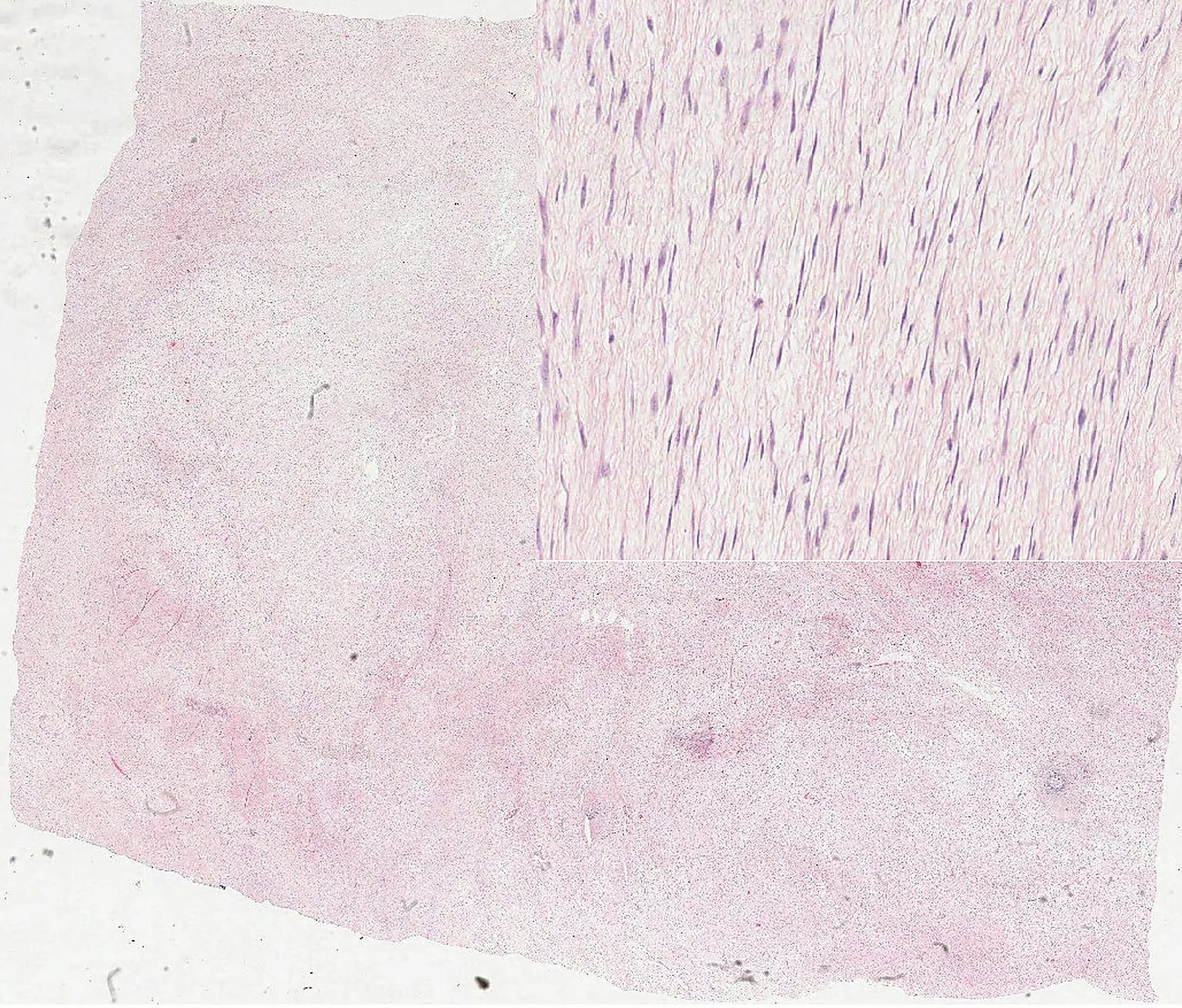
Microscopic images of resected abdominal wall desmoid Main image, original magnification ×0.4; inset, original magnification ×20.

Sporadic desmoids arise predominantly at the abdominal wall (abdominal wall desmoids (AWDs)) and extra-abdominal sites (extra-abdominal desmoids (EADs))^15^. Approximately 70% of FAP-associated desmoids occur in the abdominal cavity (intra-abdominal desmoids (IADs)), 80% of these being in the small bowel mesentery^16^ (*Figs 3* and *4*). Only 15% of FAP-associated desmoids are EADs^17^.

**Fig. 3:**
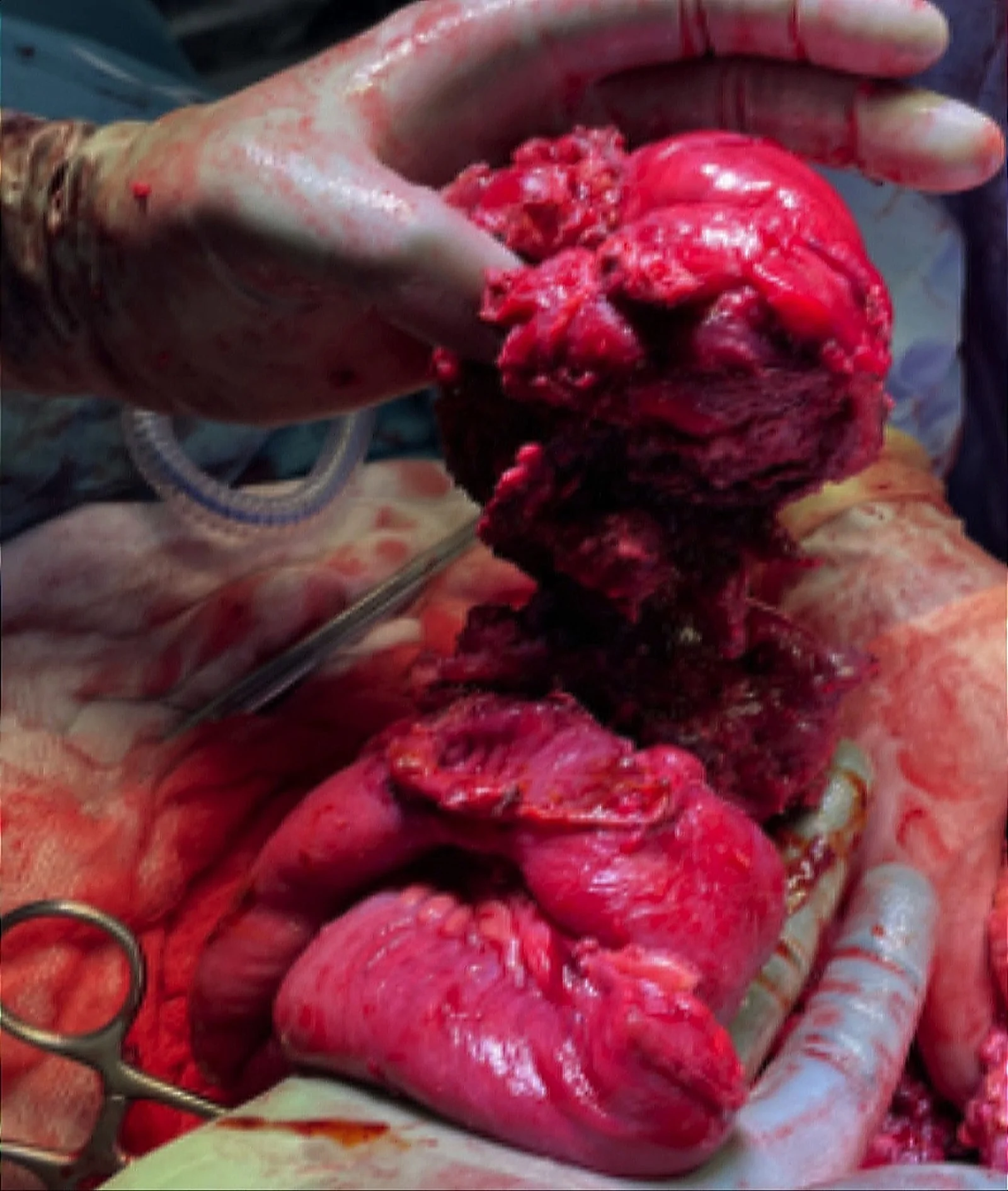
Intra-abdominal desmoid adherent to small bowel (intraoperative image)

**Fig. 4:**
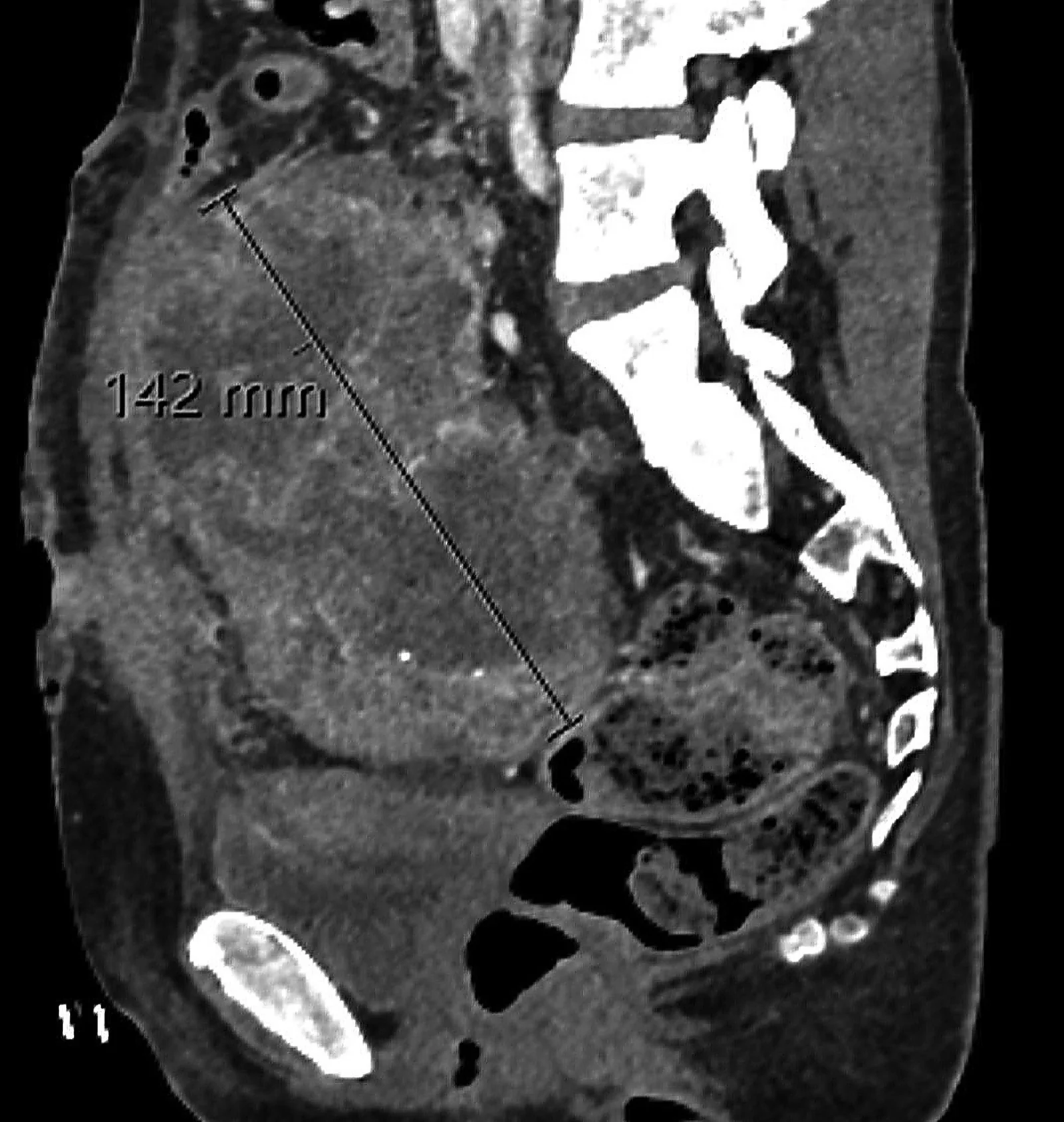
Intra-abdominal desmoid adherent to small bowel (computed tomography sagittal section)

At a molecular level, all desmoids are associated with aberrations in the Wnt/β-catenin pathway^18^. In almost all patients with sporadic desmoids, a somatic mutation is detected in *CTNNB1*, the gene that encodes for β-catenin, leading to its accumulation. Patients with FAP have a constitutional pathogenic variant in the *APC* gene, responsible for a protein that regulates β-catenin levels, resulting in the synthesis of a truncated protein that lacks normal β-catenin regulatory function, representing the initiating event in FAP-associated desmoid disease^19–25^. A (somatic) second hit then occurs in *APC*; the ‘just right’ hypothesis explains^26^ how particular combinations of constitutional and somatic variants have selective advantage and are pathological in different tissues. Consistent with the proposed mechanism of *APC* inactivation causing chromosomal instability, limited molecular characterization has demonstrated^27^ a significantly higher frequency of copy number abnormalities at the region comprising *APC* (5q22) in FAP-associated *versus* sporadic desmoids. In addition, there is some evidence of differences in microRNA expression profiles between the two, suggesting potentially distinct regulatory mechanisms at the microRNA level^27–29^.

Clinically, despite the absence of malignant features, desmoids are often locally invasive, with the potential to infiltrate adjacent tissues, compress blood vessels and nerves, erode bone, and cause bowel or ureteric obstruction and sepsis^30^. This can lead to significant morbidity and mortality^31^. FAP patients with desmoids have poor quality of life, related to uncertainty given the diagnosis and a perceived lack of available interventions. The mortality among all patients with FAP directly attributable to desmoids may be as high as 3.6%, with desmoid disease consistently reported as the second or third leading cause of death overall^32–34^.

There is sparse literature on the natural history of desmoid disease (both sporadic and FAP-associated), but the majority of FAP-associated desmoids have a disease trajectory that consists of cycles of growth and resolution, with as many as half remaining stable, one-third displaying cycles of regression and progression, and 20% resolving spontaneously^35^. An initial growth phase to a maximum size followed by spontaneous stabilization has also now been described in sporadic desmoids by a number of groups^36–39^.

### Risk factors for FAP-associated desmoids

Desmoid disease occurs most commonly in individuals in their 20s and 30s, and well established risk factors include the site of the constitutional *APC* pathogenic variant, a family history of desmoid disease, and previous trauma or surgery^40,41^. The risk conferred by these factors varies by publication, but hazard ratios (HRs) as high as 2.28–8.30 and 3.00–9.24, respectively, have been reported for high-risk pathogenic variant and family history, and vary according to approach or type of surgery for the latter^42,43^.

#### Genotype–phenotype correlation

The association between a far 3′ *APC* constitutional pathogenic variant and an increased risk of desmoid disease is well established, with the reported odds ratio (OR) for development ranging from 4.37 to 12.0^10,41^. Discrepancy remains in the literature as to the definition of a far 3′ pathogenic variant, with some authors using codon 1444 and others 1400^44^; this latter cut-off is thought to be functionally logical because it corresponds to a point immediately after the second 20-amino-acid repeat on transcription and ensures that two intact β-catenin binding repeats are retained, which has been proposed as crucial for the progression of desmoids^26,45^ (*Fig. 5*). Regardless of the precise location, a constitutional pathogenic variant 3′ of this area is clearly associated with increased risk of desmoid disease^15,44,46^.

**Fig. 5:**
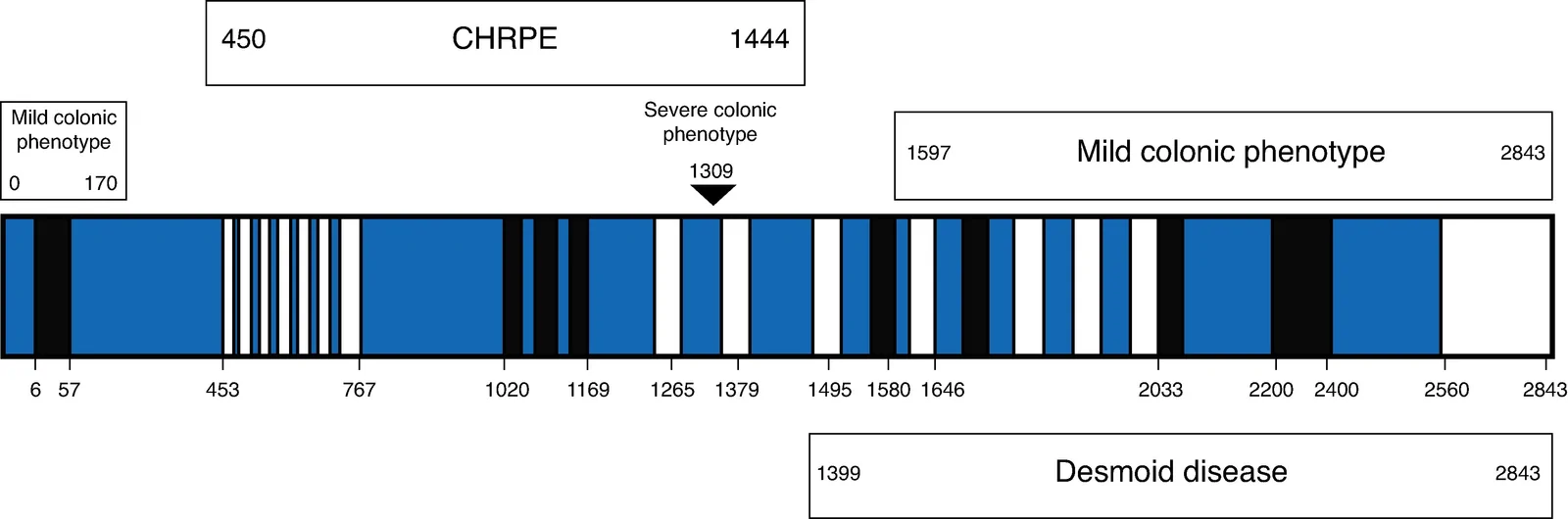
*APC* gene with genotype–phenotype correlations Mutation sites on the *APC* gene associated with different conditions. CHRPE, congenital hypertrophy of the retinal pigment epithelium.

#### Family history

A history of at least one first-degree relative with desmoid disease is a further well evidenced risk factor for desmoid formation in FAP patients, independent of pathogenic variant^10,42,45^. In fact, according to one group^41^, a positive family history was the most significant risk factor (OR 7.02). Family clustering has been demonstrated, with some published data showing that the absolute risk based on family history may be dependent on degrees of separation, suggesting a 25% risk for development if desmoid disease is present in a first-degree relative, compared with risks of 11% or 8% for second- or third-degree relatives, respectively^8^. Other work has shown that a family history of multiple desmoids increases the risk of developing multiple rather than single desmoids, suggesting additional genetic factors, such as modifier genes^47^.

#### Personal history

A personal history of a desmoid has also been shown to be associated with a substantially increased risk of subsequent (metachronous) desmoid formation in patients with FAP, with reported effect sizes ranging across cohort studies (approximate HRs 2.10–3.40)^10,48^; this risk demonstrates a clear anatomical gradient, being highest for intra-abdominal/mesenteric disease (HR 3.10), intermediate for abdominal wall lesions (HR 2.05), and lowest for extra-abdominal sites (HR 1.25–1.45), with a strong tendency for desmoids to arise at previous surgical or trauma-associated locations^46,49^.

#### Surgery

The vast majority of FAP-associated desmoids develop within 5 years after surgery, with a median time from surgery to diagnosis of 2.1–4.7 years^9,50^. The largest systematic review and meta-analysis on the subject reported an OR of 3.40 (*P* < 0.05) for desmoid development in FAP patients, in the context of a history of abdominal surgery^51^.

Most FAP patients undergo (typically by their late 20s) risk-reducing colectomy with ileorectal anastomosis or restorative proctocolectomy (RPC) with ileal pouch–anal anastomosis. Most studies have observed RPC to be associated with an increased risk of desmoid development^43,48,52–54^, with the hypothesized explanation for this being that an RPC is relatively more invasive and extensive (involving both abdominal and pelvic instrumentation and more extensive resection, as well as greater manipulation and stretching of the mesentery). With regard to different surgical approaches (that is, open *versus* minimally invasive (laparoscopic or robotic)), the data are even more heterogeneous. Some large, pooled cohorts and meta-analyses have indicated there is no difference in subsequent desmoid formation^43,51^. When looking at ileorectal anastomosis alone, the data suggest that a laparoscopic approach reduces desmoid formation^42,48^; there has been some suggestion that laparoscopic RPC is associated with greater risk than open RPC, although this remains controversial^52^.

#### Female sex

Female sex has been implicated, stemming from the observation of a higher overall incidence of sporadic desmoids in women^55^. This has been presumed to be related to oestrogen exposure, particularly given the demonstration of oestrogen receptor expression in desmoids (but in only a handful of studies)^12,56,57^, and has led to the widespread use of anti-oestrogens, also known as selective oestrogen receptor modulators. However, in FAP the data are more equivocal^15,58–60^; FAP-associated desmoid disease demonstrates only a mild female predominance (∼53–58% female; female : male ratio 1.1–1.4 : 1, often not statistically significant)^4,8,61^, in contrast to sporadic desmoids where approximately 60–75% of cases occur in women (female : male ratio 2–3 : 1)^55,62,63^. Similarly, in both the large retrospective data sets from which the other risk factors above have been quantified, the HR (1.50–3.57) for female sex falls below statistical significance^42,51^.

### Diagnosis and role of biopsy

Although for sporadic desmoids definitive diagnosis mandates biopsy of a suspected lesion, in FAP both the relatively higher underlying probability of a mass being a desmoid and the typically greater risk associated with biopsy (given the higher proportion in a mesenteric distribution and previous extensive surgery in most) mean that routine tissue diagnosis is neither necessary nor recommended^64^. Three-quarters of desmoids in FAP develop following prophylactic colonic surgery, most being IADs, and the diagnosis is usually based on compatible clinical and radiological findings in this context, with cross-sectional imaging either having been performed specifically to investigate symptoms of a possible desmoid (for example, a frank mass or obstructive symptoms) or a desmoid being an incidental finding on imaging performed for other reasons^8,41^.

Histological confirmation by biopsy is rarely required, other than in a few specific instances. The first such instance is *de novo* FAP (in the absence of a family history of FAP) where the first presentation is with a desmoid; other more common soft-tissue neoplasms need to be excluded, particularly while waiting on constitutional genetic testing. The second such instance is in the context of a previous cancer diagnosis in a patient, whereby local recurrence or metastases must be ruled out. The final, and increasingly common, scenario is when tissue confirmation is necessary to allow access to novel therapies. Clinical trials mandating a tissue diagnosis to access treatment reflects a trial design that does not distinguish between FAP and sporadic desmoids. For patients without FAP, the prior probability of a soft-tissue mass being a desmoid is very much lower and the likelihood of it being an alternative pathology much higher, and therefore biopsy is clearly indicated in this setting and usually targets a fairly superficial mass, or occasionally an intra-abdominal lesion in a virgin abdomen. Therefore, it would be helpful if future trials considered these different clinical settings in their design and did not insist on biopsy for patients with a confirmed diagnosis of FAP, no recent history of cancer, and in whom the clinical and radiological features are typical.

Where biopsy is required, fine needle aspiration and cytological assessment is prone to error, with reports of misinterpretation of malignancies^65–68^. Core needle biopsy is more appropriate and more reliable.

Recent reviews mainly focus on sporadic desmoids, which present as a rapidly growing mass in an otherwise healthy individual^69,70^. In this scenario the differential diagnosis is wide and includes lymphoma, sarcoma, and other malignancies that are far more common than desmoids. Biopsy in this setting is clearly mandatory, and allows confirmation of a sporadic desmoid if a somatic *CTNNB1* mutation is detected in the tissue.

In the pathogenesis of desmoids, the disruption of Wnt/β-catenin signalling represents a common pathway; most sporadic desmoids occur secondary to somatic alteration of the β-catenin gene (*CTNNB1*), and FAP-associated desmoids result from genetic alterations in *APC*^23^. Because somatic mutations in these two genes are mutually exclusive, the finding of a *CTNNB1* pathogenic variant essentially rules out FAP. Therefore, although the finding of wild-type *CTNNB1* in desmoid tissue is suggestive of FAP, *APC* somatic mutations causing sporadic desmoid have been reported^71^, so a full clinical work-up and constitutional genetic testing are required in this scenario.

The situation is quite different when there is a pre-existing diagnosis of FAP (especially in the years immediately after prophylactic colectomy), with the prior probability of a mass being a desmoid many orders of magnitude higher, whereas other potential diagnoses are rare. Most IADs arise after colectomy, and the resulting adhesions, and their position in the small bowel mesentery make biopsy technically difficult and the risk of bowel perforation high. In specialist centres caring for high volumes of patients with FAP (in contrast to sporadic desmoids), the diagnosis is made on the basis of clinical presentation and typical radiological features (regardless of site), and biopsy is not considered necessary. If these features are not typical, or there is a recent history of cancer (with concern for recurrence), biopsy is recommended. This approach is supported in recent guidelines^64^.

Some groups have attempted screening for asymptomatic desmoid disease. Current British Society of Gastroenterology/Association of Coloproctology of Great Britain and Ireland guidelines^72^ recommend cross-sectional imaging in patients considered high risk (that is, those with a far 3′ *APC* constitutional pathogenic variant and/or a first-degree relative with a desmoid) before prophylactic colonic surgery, with the aim of detecting asymptomatic desmoids that could impact preoperative planning. In many centres imaging is done for any patient with FAP who has had abdominal surgery and a further operation is being considered^72^. There is no evidence to support routine screening of asymptomatic patients^73^.

In a minority of patients, the FAP are AWDs and EADs, which are often more obvious than IADs, negating the need for a radiological (in favour of a clinical) diagnosis alone. When surgery is being considered, cross-sectional imaging often forms a key part of the work-up n terms of assessing resectability, as well as detecting post-operative complications and so-called recurrence^74^.

### Surveillance and imaging

Guidelines predominantly aimed at sporadic desmoid disease have shifted firmly in recent years towards an active surveillance (watch-and-wait) approach^75^. Regular cross-sectional imaging by either magnetic resonance imaging (MRI) or computed tomography (CT) for a minimum of 2 years after initial diagnosis is recommended^76^. When it comes to FAP-associated desmoid disease, there is evidence that diffusion-weighted MRI is at least as good as CT in terms of sensitivity, is better at showing the relationship to surrounding structures, and may even provide better insights into the constituents, and potentially behaviour, of the desmoids^77–84^. In addition, MRI avoids the significant accumulated radiation exposure of repeated CT^85^. Evidence is lacking at present to inform other aspects of best practice in this area, including the optimal duration of follow-up and the interval between scans.

Given the incidence of silent ureteric obstruction, 6- to 12-monthly surveillance by ultrasound for desmoid-related ureteric obstruction is a reasonable and well established practice^86^. Otherwise, the use of CT or MRI to assess suspected abdominal complications (such as small bowel obstruction and/or fistula formation) secondary to IAD is also common.

### Active surveillance and indications for treatment

Among FAP patients, most desmoids remain stable (either before or after an initial growth phase) or resolve spontaneously, with only a minority exhibiting continual progression^36–38,87^. This observation underpins the recommendation in consensus guidelines that the majority of desmoids can be managed with a watch-and-wait approach, the success of which is apparent in more recent studies, while acknowledging that a great deal of these data are from sporadic or mixed cohorts^88,89^. Ultimately, the majority of FAP-associated desmoids do not require treatment at all (and particularly not immediately or urgently). However, a minority do and, although there have been attempts to identify features predictive of aggressive growth, some of which (for example, diffusion-weighted MRI or T2-weighted imaging, or ^18^F-fluorodeoxyglucose positron emission tomography) have held promise, there is still no robust method of predicting the subset of desmoids that will grow relentlessly and do need to be treated^85,90,91^.

### Surgical management

Over the past 20 years, treatment has evolved from aggressive first-line treatment with surgery to the use of systemic agents such as non-steroidal anti-inflammatory drugs (NSAIDs) and oestrogen receptor modulators (albeit without a robust evidence base to support their use) to active surveillance as first-line management. Surgery is now reserved for symptomatic local management with a focus on best functional outcome for both FAP and sporadic desmoid disease^75,92,93^.

The latest consensus guidelines from both the Desmoid Tumour Working Group^94^ and the National Comprehensive Cancer Network^95^ recommend observation as the primary treatment option for desmoids that are not symptomatic, life threatening, or causing notable impairment. This seems a logical approach, given the high rate of cessation of growth^96,97^. Spontaneous desmoid regression has been reported in 20–55% of patients on active surveillance, with cumulative evidence of long-term stabilization^88,89,98,99^.

In the case of IADs, which comprise the majority of desmoids in FAP, although some more recent data indicate that evolving surgical techniques and careful patient selection have somewhat improved the outcomes of surgery (including a reduction in procedure-related mortality and the need for long-term parenteral nutrition), surgical excision can necessitate extensive enterectomy and be associated with considerable blood loss^100,101^. This is a result of the diffuse, infiltrative nature of the disease process, which often encases the mesenteric blood vessels. There is some anecdotal evidence that sporadic desmoids may be better circumscribed, making excision more straightforward.

There appears to be no difference in survival rates between patients having complete or partial resection^102^. These data are echoed by a systematic review^103^ reporting on outcomes of operative management in sporadic desmoids, which found that local control rates for surgery, surgery plus radiotherapy, radiotherapy alone, and active surveillance were nearly identical at around 80% and that, among patients with recurrent disease, active surveillance was associated with significantly better local control than surgery.

It is therefore generally recommended in most current guidelines^64,104^ that in IADs, surgery should be reserved for the management of complications such as intestinal obstruction, ureteric obstruction (that is, specifically unmanageable or progressive following less invasive techniques such as stenting), fistulation, necrosis, or abscess formation (with progression or recurrence despite antimicrobials and/or radiological drainage). The aim of surgery should be to resolve the complication (for example, sepsis drainage, proximal stoma formation) rather than an attempt to remove the desmoid.

The use of intestinal bypass or diversion as a non-curative surgical option in FAP-associated desmoid disease has been reported^105^ as a strategy for either managing severe complications of IADs (where invasion of surrounding structures is causing intestinal obstruction, ureteric obstruction, or enterocutaneous fistula formation) or in the situation where involvement of the mesenteric root is so extensive that radical resection would likely cause substantial bowel loss (and may even stimulate further desmoid formation); however, there are no published series on the outcomes of this approach, which is reflected in its absence from major recommendations.

EADs and AWDs, the least common sites of involvement in FAP, tend to cause mortality less frequently and are typically (although not universally) more easily accessible for surgery without disruption of critical structures, which formed the basis of historical recommendations that they can be safely excised, and that surgery should be considered first-line. However, more recent consensus guidelines^76^ have been unequivocal in their updated algorithms and advise that surgery is no longer the initial/default treatment for EAD or AWDs; instead, surgery should be reserved for the minority of symptomatic patients in whom there is a clear functional deficit (or pain) that requires active intervention, and be based on individual patient factors and specific desmoid behaviour. Similarly, it is chiefly in this same clinical context that recommendations suggest consideration of one of the many non-surgical local control strategies, such as radiofrequency ablation, cryoablation, high-intensity focused ultrasound, and chemoembolization, among others^106–111^.

Finally, there is a discussion about the recurrence of desmoid disease, with most literature describing local recurrence rates in the range of 20–40%^112^. However, uniquely, compared with other soft tissue neoplasms, it is difficult to distinguish whether these figures reflect true recurrence, whether they represent cases of incompletely resected desmoids (because margins can be difficult to distinguish), or whether they are entirely new desmoids precipitated by the surgical trauma of the excision itself^112^. This distinction is reflected in a recently proposed set of treatment algorithms^70^ appropriately distinguishing AWDs (both sporadic and FAP-associated; for which surgery (and local ablative techniques, such as radiofrequency ablation and cryotherapy) is placed alongside medical therapy as a potential primary treatment option) from mesenteric desmoids (for which medical therapy is positioned as the first-line treatment strategy, whereas surgery is advised exclusively in the scenario that ‘expected morbidity is low’ for sporadic desmoids or ‘only to treat complications’ for FAP-associated desmoids).

### Medical management

Most FAP-associated desmoids seem to stop growing (or even regress spontaneously), so many patients can be managed conservatively with active surveillance. However, should desmoids progress, systemic pharmacotherapy is advised as the first-line treatment strategy. This avoids the morbidity of surgery in the setting of likely infiltrative margins, deep location, or invasion of critical structures, as well as being the logical choice where disease is multifocal.

In the landscape of a rare disease and historic small, poor-quality studies (often lacking a control arm), only a single agent to date has received formal regulatory approval (specifically, by the United States Food and Drug Administration as well as the European Medicines Agency) for the treatment of desmoids, underlining the challenge of this strategy.

Although there have been no randomized clinical trials (RCTs) evaluating its use, cytotoxic chemotherapy has been used to treat desmoid disease (sporadic and FAP-associated), with reported clinical response in over three-quarters of patients^113^. Drug combinations are mainly based on vinorelbine, vinblastine, methotrexate, or (liposomal) doxorubicin^114^. In FAP-associated desmoid disease, two main regimens have been used. The first is effectively a sarcoma regimen, consisting of doxorubicin and dacarbazine, for which some evidence of efficacy has existed since the 1990s^115,116^. One case series included 7 patients with a 43% partial response rate and a 57% complete response (CR) rate^117^. A larger multicentre observational study from Japan using the same combination reported a 40% overall response rate (ORR) and an 80% disease control rate (DCR), in absence of major adverse events (AEs)^114^. Another widely used regimen is methotrexate plus a vinca alkaloid (usually vinblastine or vinorelbine). This was also reported on in the Japanese study^114^, with a 67% ORR, whereas another more recent multicentre retrospective cohort study^118^, consisting of 37 patients with FAP-associated desmoids, reported a DCR of 95% (with no associated mortality or grade 3 or 4 toxicity). Otherwise, for sporadic desmoids, a chemotherapy regimen consisting of low-dose methotrexate in combination with a vinca alkaloid has also been used with some apparent success^119^. Finally, several more recent retrospective studies^120,121^ have reported on single-agent oral vinorelbine, demonstrating a DCR as high as 86% alongside symptomatic improvement and possibly less toxicity (although comparative studies have not been conducted).

Although the precise mechanism of action of these agents is not clear, there has been gradually increasing interest in newer so-called molecular-targeted therapies in treating desmoid disease. The first such agents were tyrosine kinase inhibitors (TKIs)^122^. Imatinib is a c-KIT TKI used in the treatment of gastrointestinal stromal tumours (with inhibition of platelet-derived growth factor receptor β being postulated as the mechanism of action in desmoid disease)^123,124^. Multiple prospective trials^125,126^ have evaluated the safety and efficacy of TKIs in mixed desmoid populations and reported apparent favourable outcomes, even in the context of progression on previous chemotherapy. In one study, in which 19 patients with progressive desmoids (ten sporadic and nine FAP-associated) received imatinib, the CR rate was 16% and the DCR was 84%^127^.

A phase III RCT focusing primarily on sporadic desmoids (and not specifically reporting the number of patients with FAP enrolled or *APC* mutation status) found that sorafenib significantly improved progression-free survival compared with placebo^35^. The 2-year progression-free survival rate was 81% for sorafenib, compared with 36% for placebo, and the study was halted early due to the positive results, with placebo patients allowed to cross over to the sorafenib group. This work established sorafenib as an effective therapy for this rare tumour type^35^. Pazopanib, another TKI currently in use in this setting (and licenced for use in sarcomas) has a number of targets (vascular endothelial growth factor receptors 1, 2, and 3; platelet-derived growth factor receptors α and β; and c-KIT tyrosine kinases) and has also demonstrated superior progression-free survival and objective response rate compared with chemotherapy (with methotrexate and vinblastine) in an RCT setting^125^. In conclusion, TKIs are seemingly effective, convenient, and have a relatively acceptable AE profile (permanent hypertension or thyroid dysfunction, being the only notable exceptions), broadly supporting their increasing use as systemic therapy in desmoids^128^.

Nirogacestat, the only drug with formal licenced regulatory approval for use in treating desmoid disease, is a γ-secretase inhibitor. Dysregulation of cross-talk between the Notch and Wnt/β-catenin signalling pathways is implicated in tumourigenesis, progression, and treatment resistance in desmoid disease; the mechanism of action of the γ-secretase inhibitor is to block Notch receptor proteolysis and subsequent translocation of the Notch intracellular domain to the nucleus^129^. Despite its only relatively recent use and approval, this agent’s licence is well founded on the basis of clinical trial data, including a phase III international double-blind randomized placebo-controlled trial involving 142 patients^130^, which reported a higher objective response rate (41% (95% confidence interval (c.i.) 29.8 to 53.8) *versus* 8% (95% c.i. 3.1 to 17.83)) and CR rates (7% *versus* 0%) compared with placebo. However, although nirogacestat may be an important new systemic therapy, the proportion of patients with FAP-associated desmoid disease was very modest (29 of 142, 20%), with only 13 of these patients receiving the drug (objective response rate 38% *versus* 13% with placebo)^131^.

Historically, NSAIDs and anti-oestrogens (also known as oestrogen receptor modulators) were often used to treat desmoids, but no RCT of either drug class has been conducted in this context. The evidence on which their use was based consisted of case reports and small uncontrolled series, most of which document outcomes little different from untreated desmoid disease. NSAIDs and anti-oestrogens are now falling out of regular use, in favour of different pharmacological approaches outlined above, and this transition is evident in the most recent consensus guidelines^76^.

The original rationale for NSAID use was the observation that cyclo-oxygenase (COX)-2 is overexpressed in desmoid tissue^132^. NSAIDs that inhibit both COX-1 and COX-2 (sulindac and indomethacin) and selective COX-2 inhibitors (such as celecoxib) have been investigated in the treatment of desmoids, including FAP-associated desmoid disease^133,134^. Overall, however, among what are effectively entirely observational studies, both wide ranges of response and variable tolerability are reported^73,135–138^. In the case of FAP-associated desmoid disease, the quality of data are diminished further by small patient numbers, with all but two published studies including fewer than 30 patients. Hence, although NSAIDs (particularly sulindac) have been used widely and historically in treating desmoid disease, their use as disease-modifying agents is unsupported.

The use of anti-oestrogens (tamoxifen, toremifene, and the second-generation selective oestrogen receptor modulator raloxifene) arose from a number of observations, including a greater incidence of sporadic desmoids in women and a potential higher desmoid growth rate in pregnancy, and the identification of female sex as a possible risk factor for desmoid disease in FAP^60,62,63^. Once again, however, published studies consist entirely of small retrospective observational data sets. Thus, despite their previously ubiquitous use, the improved long-term safety profile among newer agents, and some encouraging reported outcomes in small series, the evidence-base for anti-oestrogens is currently insufficient to justify their ongoing use^137,139–141^.

At present the only agents for which RCT data support use as systemic therapy in FAP-associated desmoid disease are the TKIs and the γ-secretase inhibitor nirogacestat. Importantly, however, confounding all of the published studies (including those RCTs) is the presence of mixed desmoid populations, resulting in very small numbers of patients with FAP. It is also worth highlighting that all these studies use imaging-derived endpoints based on RECIST (Response in Oncologic Imaging of Solid Tumours) criteria to define their outcomes, which are based solely on changes in maximum tumour diameter and may simply not even identify all instances of clinical response, let alone quantify them^84,142,143^.

### Intestinal transplantation

The treatment of IADs resulting in significant symptoms or complications despite systemic therapy (or when such therapy is contraindicated by sepsis, for example) has been by excision^70,104^. Such surgery is often technically challenging due to involvement of adjacent critical structures (the bowel and its blood supply, major vessels, ureters, bladder, or abdominal wall) compounded by an often septic or malnourished patient^102^. Achieving adequate excision may be impossible without a total or near-total enterectomy, resulting in short bowel syndrome and intestinal failure, with potential life-long dependence on parenteral nutrition^144^.

Intestinal transplantation is increasingly recognized as a treatment option for intestinal failure due to desmoid disease when conventional therapy has been unsuccessful (that is, the disease cannot be safely treated surgically but is symptomatic) or when surgery has resulted in intestinal failure. This is particularly useful in the context of complications limiting the use of parenteral nutrition, such as venous parenteral nutrition-associated liver disease, or near depletion of venous access^145^.

Intestinal transplantation, either isolated intestinal or multivisceral, can potentially offer complete treatment of desmoid disease for some patients, without compromising intestinal function. Synchronous transplantation of an abdominal wall flap can allow management of significant abdominal wall defects without requiring mobilization of flaps from elsewhere in the patient (and risk of desmoid disease occurring at the site due to trauma).

Although this has yet to be adopted outside a handful of high-volume specialist centres, intestinal transplantation with specific inclusion of a foregut graft can offer a risk-reduction strategy in FAP patients with advanced luminal disease of the upper gastrointestinal tract that is unmanageable endoscopically^146,147^. In these circumstances, prophylactic duodenectomy, gastrectomy, or both are offered to prevent cancer, but can be rendered impossible by the presence of small bowel mesenteric desmoid disease^146,147^.

The possibility of developing further or recurrent desmoid disease after small bowel transplantation (an extensive surgical insult) warrants caution. In one series, AWD occurred in two of nine patients with FAP after small bowel transplantation for IAD, whereas no IAD recurrence was observed in another patient, although three patients developed EADs elsewhere after the transplant^148^. Furthermore, the need to achieve total clearance remains controversial, with conflicting literature regarding its impact on desmoid disease recurrence rates^149,150^.

Intestinal transplantation requires long-term immunosuppression, and is associated with a risk of opportunistic infections, the possibility of rejection, and a lack of availability of donor organs. One group has described partial abdominal evisceration, *ex vivo* desmoid resection, and autotransplantation as a possible solution to the lack of availability of donor organs^151^.

In terms of detailed outcomes, although earlier series established the feasibility of intestinal and multivisceral transplantation for FAP-associated desmoid disease^148,151–153^, more contemporary reports demonstrate progressively improving outcomes in specialized centres^146,147,154,155^, with acute rejection occurring in approximately 30–50% of recipients but typically responsive to immunosuppression, and overall morbidity remaining high yet largely manageable. Recent series^146,147,155^ also report encouraging survival outcomes, with 5-year patient survival ranging from 80% to 92% and 10-year survival up to around 80%, alongside prolonged but acceptable median hospital stays (often several weeks) and frequent but anticipated readmissions related to infection or graft surveillance. These concerns and criticisms aside, the preliminary early outcomes for patients are therefore overall promising with respect to both graft function and survival outcomes.

### Future directions and evidence gaps

Progress has been made in terms of bringing a more rational approach to the management of desmoid disease, given the limited evidence base. Some key areas remain where further research would have significant impact.

Given that only a minority of desmoids progress to cause major morbidity and mortality and that a watch-and-wait approach to initial management is advocated, evidence is needed to inform choice of imaging modality and frequency, as well as to identify features predictive of continuing growth. This would allow identification and timely treatment of aggressive desmoids, and prevent overinvestigation and treatment of the majority of desmoids that will stabilize.

Imaging plays a crucial role in the management of desmoid disease in FAP. Beyond initial diagnosis, this spans active surveillance and assessment of progression and response to systemic treatment^80,156,157^. However, there remains a lack of evidence (and resultant diversity in approach) in almost all aspects of practice in this area.

Several classification systems have been proposed for FAP-associated desmoid disease^157–159^. Although some groups do base their management choices on such classification systems, none of these has been widely adopted^160^. Another important issue in the area of imaging is that study endpoints that are used to assess the response to pharmacotherapy are virtually all based on RECIST criteria, despite this arguably being inappropriate when assessing a non-malignant entity that can grow in plaque-like sheets^84,143^. Except for the recent DeFi trial (which defined symptomatic response as a key outcome), if currently used imaging-based outcome measures do not fully capture clinically meaningful treatment response, then effective clinical trials cannot be designed and the validity of existing drug studies is potentially called into question^130^.

Although progress has been made on pharmacotherapy, at the time of writing only a single agent (nirogacestat) has received regulatory approval for use in this condition, and it is the only agent to have been subject to an RCT (with imaging-based endpoints). Only one other class of medications (the TKIs) has undergone testing with either a control arm or some degree of randomization^130^. In addition, these studies comprise mixed cohorts encompassing both sporadic and FAP-associated patients, which form a minority.

Finally, most desmoids in FAP seem to be triggered by the trauma of prophylactic colectomy, and a subgroup at particularly high risk is well defined. This raises the possibility of a chemoprevention strategy, which could be implemented around the time of prophylactic surgery, with the aim of reducing subsequent desmoid formation. Consequently, uncertainty remains regarding the magnitude of benefit, specifically in genetically defined FAP-associated disease.

Desmoid disease is an enigmatic and poorly understood disease entity. Because the vast majority of desmoids are sporadic, presenting as possible soft-tissue malignancy, and because most of the drugs used in treatment are also used in cancer therapy, most desmoids are managed by sarcoma oncologists and surgeons. This has resulted in the ubiquitous use of cancer terminology; in addition to being flawed and inaccurate for a non-malignant, often self-limiting process like desmoid disease, the use of RECIST criteria, alongside terms such as ‘recurrence’ and ‘resection margins’, all of which carry the connotations of a malignant neoplasm, serves to reinforce potentially inappropriate practice (such as unnecessary biopsy, the use of unsuitable trial endpoints during pharmaceutical studies, or striving for wide resection margins during surgery).

There is undoubted biological and clinical distinction between sporadic and FAP-associated desmoid disease, and the context of the other manifestations and surgery needed in FAP add a further dimension to this. Data from trials on sporadic desmoids should be extrapolated cautiously to FAP-associated disease.

## Supplementary Material

zrag089_Supplementary_Data

## Data Availability

This article is based on previously published studies. No new data were generated or analysed, and data sharing is not applicable.
