# Supplementary material for: Familial adenomatous polyposis-associated desmoid disease: comprehensive review
Source: BJS Open. 2026 Sep 29;10(5):zrag089. doi: 10.1093/bjsopen/zrag089 (PMC13621219; doi:10.1093/bjsopen/zrag089)
Supplement: Supplementary file 1 [file zrag089_supplementary_data.docx]

**Familial adenomatous polyposis-associated desmoid disease: comprehensive review**

Benjamin Zare^1,2*^, Susan Clark^1,2^, Palma Dileo^3,4^, David Liska^5,6^, Andrew Latchford^1,2^

Affiliations:

1. St Mark’s Centre for Familial Intestinal Cancer, St Mark's Hospital, London, UK.
2. Department of Surgery and Cancer, Imperial College London, London, UK.
3. University College London Hospitals NHS Foundation Trust, London, UK.
4. Istituto Ortopedico Rizzoli, Bologna, Italy.
5. Department of Colon and Rectal Surgery, Digestive Disease and Surgery Institute, Cleveland Clinic Foundation, Ohio, USA
6. The Sanford R. Weiss, MD Center for Hereditary Colorectal Neoplasia, Department of Colorectal Surgery, Digestive Disease Institute, Cleveland Clinic, Ohio, USA.

^*^Corresponding author:

Please submit all correspondence and requests for reprints to

Benjamin Zare

Email: b.zare@nhs.net

Address: St Mark’s Hospital, Acton Lane, London. NW10 7NS

**SUPPLEMENTARY MATERIAL**

**Supplementary Figures and Tables**

| S1 Search strategy. | Page 3 |
| --- | --- |
| S2 PRISMA-ScR Table. | Page 6 |

**S1. Search strategy**

Searches were undertaken in accordance with PRISMA methodology. The search was designed to identify original research studies, reviews and guidelines relating to desmoid disease, FAP-associated desmoid disease and familial adenomatous polyposis published up to August 2025. Non-English language publications were excluded. Data were extracted using the JBI data extraction tool and synthesised narratively by topic.

**MEDLINE (OVID)**

1. exp Fibromatosis, Aggressive/ or exp Fibromatosis/

2. (desmoid* or "desmoid tumour*" or "desmoid tumor*" or "desmoid-type fibromatosis" or "aggressive fibromatosis" or "deep fibromatosis").ti,ab,kf.

3. 1 or 2

4. exp Adenomatous Polyposis Coli/ or exp Adenomatous Polyposis/ or exp Familial Adenomatous Polyposis/

5. ("familial adenomatous polyposis" or FAP or "adenomatous polyposis coli" or APC).ti,ab,kf.

6. 4 or 5

7. 3 and 6

8. limit 7 to English language

9. limit 8 to yr="1946 - 2025"

**EMBASE (OVID)**

1. exp desmoid tumor/ or exp fibromatosis/

2. (desmoid* or "desmoid tumour*" or "desmoid tumor*" or "desmoid-type fibromatosis" or "aggressive fibromatosis" or "deep fibromatosis").ti,ab,kw.

3. 1 or 2

4. exp familial adenomatous polyposis/ or exp adenomatous polyposis coli/ or exp APC gene/

5. ("familial adenomatous polyposis" or FAP or "adenomatous polyposis coli" or APC).ti,ab,kw.

6. 4 or 5

7. 3 and 6

8. limit 7 to English language

9. limit 8 to (embase and yr="1974 - 2025")

**PROSPERO**

1. desmoid

2. desmoid tumour OR desmoid tumor OR desmoid-type fibromatosis OR aggressive fibromatosis

3. familial adenomatous polyposis OR FAP OR adenomatous polyposis coli OR APC

4. 1 OR 2

5. 3 AND 4

6. Records published or registered up to August 2025 were considered.

**COCHRANE**

1. MeSH descriptor: [Fibromatosis, Aggressive] explode all trees

2. (desmoid* or "desmoid tumour*" or "desmoid tumor*" or "desmoid-type fibromatosis" or "aggressive fibromatosis" or "deep fibromatosis"):ti,ab,kw

3. #1 or #2

4. MeSH descriptor: [Adenomatous Polyposis Coli] explode all trees

5. ("familial adenomatous polyposis" or FAP or "adenomatous polyposis coli" or APC):ti,ab,kw

6. #4 or #5

7. #3 and #6

8. Trials, reviews and protocols published up to August 2025 were considered.

**CAMPBELL COLLABORATION**

1. desmoid OR "desmoid tumour" OR "desmoid tumor" OR "desmoid-type fibromatosis" OR "aggressive fibromatosis"

2. "familial adenomatous polyposis" OR FAP OR "adenomatous polyposis coli" OR APC

3. 1 AND 2

4. Reviews and evidence syntheses published up to August 2025 were considered.

**JOANNA BRIGGS INSTITUTE (JBI) EVIDENCE SYNTHESIS**

1. desmoid OR "desmoid tumour" OR "desmoid tumor" OR "desmoid-type fibromatosis" OR "aggressive fibromatosis" OR "deep fibromatosis"

2. "familial adenomatous polyposis" OR FAP OR "adenomatous polyposis coli" OR APC

3. 1 AND 2

4. JBI Evidence Synthesis records published up to August 2025 were considered.

**Eligibility Criteria**

| **Inclusion criteria** | **Exclusion criteria** |
| --- | --- |
| 1. Original research studies, reviews and guidelines relating to desmoid disease, FAP-associated desmoid disease or familial adenomatous polyposis.  2. Human clinical, translational, genetic, radiological, pathological, surgical or pharmacological studies relevant to FAP-associated desmoid disease.  3. Publications available in English.  4. Publications available up to August 2025. | 1. Non-English language publications.  2. Publications not relating to desmoid disease, FAP-associated desmoid disease or familial adenomatous polyposis.  3. Non-human studies without direct relevance to human disease.  4. Conference abstracts or records without sufficient retrievable information for narrative synthesis. |

**Data charting process and outcome measures**

1. Pathogenesis and molecular biology of FAP-associated desmoid disease, including APC-associated Wnt/beta-catenin signalling.
2. Epidemiology, anatomical distribution and natural history of FAP-associated desmoid disease.
3. Risk factors including *APC* genotype, family history, personal desmoid history, abdominal surgery and sex.
4. Diagnosis, role of biopsy, *CTNNB1*/*APC* testing considerations and differential diagnosis.
5. Surveillance and imaging strategies, including CT, MRI, diffusion-weighted MRI and monitoring for ureteric obstruction.
6. Active surveillance and indications for treatment.
7. Surgical, local ablative and systemic treatment options, including NSAIDs, anti-oestrogens, chemotherapy, tyrosine kinase inhibitors and nirogacestat.
8. Intestinal transplantation for advanced intra-abdominal desmoid disease and intestinal failure.
9. Future research priorities and evidence gaps relevant to genetically defined FAP-associated desmoid cohorts.

**S2 Preferred Reporting Items for Systematic reviews and Meta-Analyses extension for Scoping Reviews (PRISMA-ScR) Checklist**

| **SECTION** | **ITEM** | **PRISMA-ScR CHECKLIST ITEM** | **REPORTED ON PAGE #** |
| --- | --- | --- | --- |
| TITLE | Title 1 | Identify the report as a scoping review. | Title identifies the article as a comprehensive review: page 1. |
| ABSTRACT | Structured summary 2 | Provide a structured summary that includes (as applicable): background, objectives, eligibility criteria, sources of evidence, charting methods, results, and conclusions that relate to the review questions and objectives. | Page 2. |
| INTRODUCTION | Rationale 3 | Describe the rationale for the review in the context of what is already known. Explain why the review questions/objectives lend themselves to a scoping review approach. | Page 4. |
| INTRODUCTION | Objectives 4 | Provide an explicit statement of the questions and objectives being addressed with reference to their key elements or other relevant key elements used to conceptualize the review questions and/or objectives. | Page 4. |
| METHODS | Protocol and registration 5 | Indicate whether a review protocol exists; state if and where it can be accessed; and if available, provide registration information, including the registration number. | Not applicable: no registered review protocol is reported. PROSPERO was searched as an information source. |
| METHODS | Eligibility criteria 6 | Specify characteristics of the sources of evidence used as eligibility criteria and provide a rationale. | Abstract page 2; Methods page 4; Supplementary Material S1. |
| METHODS | Information sources^*^ 7 | Describe all information sources in the search, as well as the date the most recent search was executed. | Abstract page 2; Methods page 4; Supplementary Material S1. Searches covered Medline, Embase, PROSPERO, Cochrane, Campbell and JBI databases up to August 2025. |
| METHODS | Search 8 | Present the full electronic search strategy for at least 1 database, including any limits used, such that it could be repeated. | Supplementary Material S1. |
| METHODS | Selection of sources of evidence^†^ 9 | State the process for selecting sources of evidence included in the scoping review. | Methods page 4; Supplementary Material S1. |
| METHODS | Data charting process^‡^ 10 | Describe the methods of charting data from the included sources of evidence and any processes for obtaining and confirming data from investigators. | Methods page 4; Supplementary Material S1. |
| METHODS | Data items 11 | List and define all variables for which data were sought and any assumptions and simplifications made. | Methods page 4; Supplementary Material S1. |
| METHODS | Critical appraisal of individual sources of evidence^§^ 12 | If done, provide a rationale for conducting a critical appraisal of included sources of evidence; describe the methods used and how this information was used in any data synthesis. | Not applicable: formal pre-structured critical appraisal was not undertaken; relevance and reliability of pertinent articles are considered narratively throughout the review. |
| METHODS | Synthesis of results 13 | Describe the methods of handling and summarizing the data that were charted. | Abstract page 2; Methods page 4; findings synthesised narratively by topic. |
| RESULTS | Selection of sources of evidence 14 | Give numbers of sources of evidence screened, assessed for eligibility, and included in the review, with reasons for exclusions at each stage, ideally using a flow diagram. | Not reported: no PRISMA flow diagram or numerical screening summary is included for this comprehensive narrative review. |
| RESULTS | Characteristics of sources of evidence 15 | For each source of evidence, present characteristics for which data were charted and provide the citations. | Thematic evidence summary pages 5-22; references pages 23-39. |
| RESULTS | Critical appraisal within sources of evidence 16 | If done, present data on critical appraisal of included sources of evidence. | Not applicable. |
| RESULTS | Results of individual sources of evidence 17 | For each included source of evidence, present the relevant data that were charted that relate to the review questions and objectives. | Thematic results and narrative synthesis pages 5-22; references pages 23-39. |
| RESULTS | Synthesis of results 18 | Summarize and/or present the charting results as they relate to the review questions and objectives. | Pages 5-22. |
| DISCUSSION | Summary of evidence 19 | Summarize the main results, link to the review questions and objectives, and consider the relevance to key groups. | Pages 20-22; abstract conclusion page 2. |
| DISCUSSION | Limitations 20 | Discuss the limitations of the scoping review process. | Evidence gaps and limitations of the available literature are discussed on pages 20-22. |
| DISCUSSION | Conclusions 21 | Provide a general interpretation of the results with respect to the review questions and objectives, as well as potential implications and/or next steps. | Abstract conclusion page 2; future directions and evidence gaps pages 20-22. |
| FUNDING | Funding 22 | Describe sources of funding for the included sources of evidence, as well as sources of funding for the scoping review. Describe the role of the funders of the scoping review. | Funding statement page 1. |

JBI = Joanna Briggs Institute; PRISMA-ScR = Preferred Reporting Items for Systematic reviews and Meta-Analyses extension for Scoping Reviews.

^*^ Where sources of evidence are compiled from, such as bibliographic databases, social media platforms, and Web sites.

^†^ A more inclusive/heterogeneous term used to account for different types of evidence or data sources that may be eligible in a scoping review as opposed to only studies.

^‡^ The frameworks by Arksey and O’Malley and Levac and colleagues and the JBI guidance refer to the process of data extraction in a scoping review as data charting.

^§^ The process of systematically examining research evidence to assess its validity, results, and relevance before using it to inform a decision.

From: Tricco AC, Lillie E, Zarin W, O'Brien KK, Colquhoun H, Levac D, et al. PRISMA Extension for Scoping Reviews (PRISMA-ScR): Checklist and Explanation. Ann Intern Med. 2018;169:467-473. doi: 10.7326/M18-0850
